# Dopamine D2 Receptor Stimulation Potentiates PolyQ-Huntingtin-Induced Mouse Striatal Neuron Dysfunctions via Rho/ROCK-II Activation

**DOI:** 10.1371/journal.pone.0008287

**Published:** 2009-12-15

**Authors:** Carole Deyts, Beatriz Galan-Rodriguez, Elodie Martin, Nicolas Bouveyron, Emmanuel Roze, Delphine Charvin, Jocelyne Caboche, Sandrine Bétuing

**Affiliations:** 1 CNRS UMR 7102, Université Pierre et Marie Curie-Paris 6, Paris, France; 2 INSERM UMRS 952, CNRS UMR 7224, Université Pierre et Marie Curie-Paris 6, Paris, France; 3 Biological Sciences Division, University of Chicago, Chicago, Illinois, United States of America; 4 Service de Neurologie, Hôpital Salpêtrière, Assitance Publique-Hôpitaux de Paris, Paris, France; 5 Université Evry Val d'Essonne, Evry, France; University of Nebraska, United States of America

## Abstract

**Background:**

Huntington's disease (HD) is a polyglutamine-expanded related neurodegenerative disease. Despite the ubiquitous expression of expanded, polyQ-Huntingtin (ExpHtt) in the brain, striatal neurons present a higher susceptibility to the mutation. A commonly admitted hypothesis is that Dopaminergic inputs participate to this vulnerability. We previously showed that D2 receptor stimulation increased aggregate formation and neuronal death induced by ExpHtt in primary striatal neurons in culture, and chronic D2 antagonist treatment protects striatal dysfunctions induced by ExpHtt in a lentiviral-induced model system in vivo. The present work was designed to elucidate the signalling pathways involved, downstream D2 receptor (D2R) stimulation, in striatal vulnerability to ExpHtt.

**Methodology/Principal Findings:**

Using primary striatal neurons in culture, transfected with a tagged-GFP version of human exon 1 ExpHtt, and siRNAs against D2R or D1R, we confirm that DA potentiates neuronal dysfunctions via D2R but not D1R stimulation. We demonstrate that D2 agonist treatment induces neuritic retraction and growth cone collapse in Htt- and ExpHtt expressing neurons. We then tested a possible involvement of the Rho/ROCK signalling pathway, which plays a key role in the dynamic of the cytoskeleton, in these processes. The pharmacological inhibitors of ROCK (Y27632 and Hydroxyfasudil), as well as siRNAs against ROCK-II, reversed D2-related effects on neuritic retraction and growth cone collapse. We show a coupling between D2 receptor stimulation and Rho activation, as well as hyperphosphorylation of Cofilin, a downstream effector of ROCK-II pathway. Importantly, D2 agonist-mediated potentiation of aggregate formation and neuronal death induced by ExpHtt, was totally reversed by Y27632 and Hydroxyfasudil and ROCK-II siRNAs.

**Conclusions/Significance:**

Our data provide the first demonstration that D2R-induced vulnerability in HD is critically linked to the activation of the Rho/ROCK signalling pathway. The inclusion of Rho/ROCK inhibitors could be an interesting therapeutic option aimed at forestalling the onset of the disease.

## Introduction

Huntington's disease (HD), a neurodegenerative disorder characterized by motor, cognitive, and psychiatric disorders (1) is caused by abnormal expansion of a CAG tract in exon 1 of the *IT15* gene. This mutation leads to an abnormal polyglutamine expansion in the N-terminal part of the huntingtin (Htt) protein. Cleavage of polyQ expanded huntingtin (ExpHtt) by caspases, leads to the release of N-terminal fragments containing the polyglutamine repeats, which can aggregate in neurites, cytoplasm, and nuclei.

Despite ubiquitous expression of Htt throughout the brain and other tissues, medium spiny GABAergic neurons in the striatum predominantly degenerate in the brain of HD patients [1]. Among various mechanisms [2] dopamine (DA) may be important in this preferential vulnerability. HD neuropathology progresses in the striatum according to the same dorsoventral gradient as local DA concentration [1], [3], [4]. Variations of DA concentration can modulate striatal death in various models [5], [6], [7]. Dopamine transporter knock-out (DAT-/-) mice display both spontaneous striatal death and behavioral alterations that resemble HD [8]. When mated to knock-in HD mice these mice showed exacerbation of HD pathophysiology and acceleration of aggregate formation [9]. We recently extended these observations and demonstrated, *in vitro*, that DA accelerates two neuropathological hallmarks induced by expHtt: aggregate formation and striatal neuron death [10]. On this in vitro model system, DA exerted a dual role on striatal death, via two independent pathways. One was due to reactive oxygen species and activation of the pro-apoptotic cJun-N terminal Kinase (JNK) pathway, the other one was specifically linked to D2 receptor stimulation and aggregate formation. Importantly, D2 antagonist treatments *in vitro* and *in vivo* protected from aggregate formation and striatal dysfunctions induced by ExpHtt [10], [11].

Aggregate formation is known to be involved in the destabilization of microtubules and disorganization of the dendritic arbors, which are early events in the pathogenic mechanisms involved in HD [12], [13], [14], [15], [16]. One critical intracellular signalling pathway involved in actin cytoskeleton rearrangements and neurites elongation is the Rho/ROCK pathway [17], [18], [19]. This pathway has recently been shown to be involved in aggregate formation and cell death induced by ExpHtt, in drosophila or cell line models [20], [21], [22]. In the present study, we show a coupling of D2 receptor to this signalling pathway. The inhibition of ROCK activity using selective inhibitors, of knock-down of ROCK-II expression reversed D2 agonist-mediated aggregate formation, neuritic retraction and neuronal death induced by ExpHtt. By contrast, these treatments failed to affect neuronal dysfunctions induced by ExpHtt itself. We thus conclude that striatal neurons vulnerability in HD may be at least in part, mediated by the Rho/ROCK signalling pathway. The inclusion of Rho/ROCK inhibitors could be an interesting therapeutic option aimed at forestalling the onset of the disease.

## Results

### DA-Mediated Potentiation of Aggregates Formation and Neuronal Death Induced by ExpHtt in Striatal Neurons Involved D2R but Not D1R Stimulation

**Figure 1:**
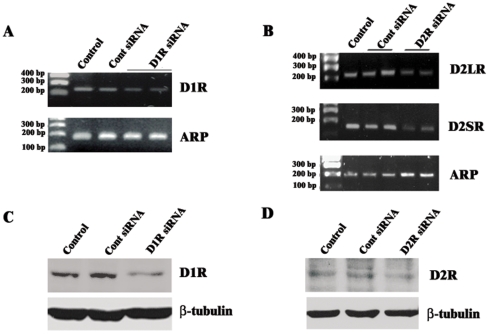
Validation of D1R and D2R specific siRNA in primary striatal neurons. A–D: primary striatal neurons were transfected or not (Control) with Cont siRNA, D1R siRNA and D2R siRNA. After 72 hours, mRNAs were extracted followed by RT-PCR (A and B) and protein expression levels detected by immunoblot (C and D). A: RT-PCR of D1R gene and, B: RT-PCR D2R. Note the presence of both D2R long and short isoforms. For A and B: ARP gene was used as an housekeeping gene. B and D: immunoblot using specific anti-D1R (C), and specific D2R (D) antibodies. β-tubulin was used for control loading.

**Figure 2:**
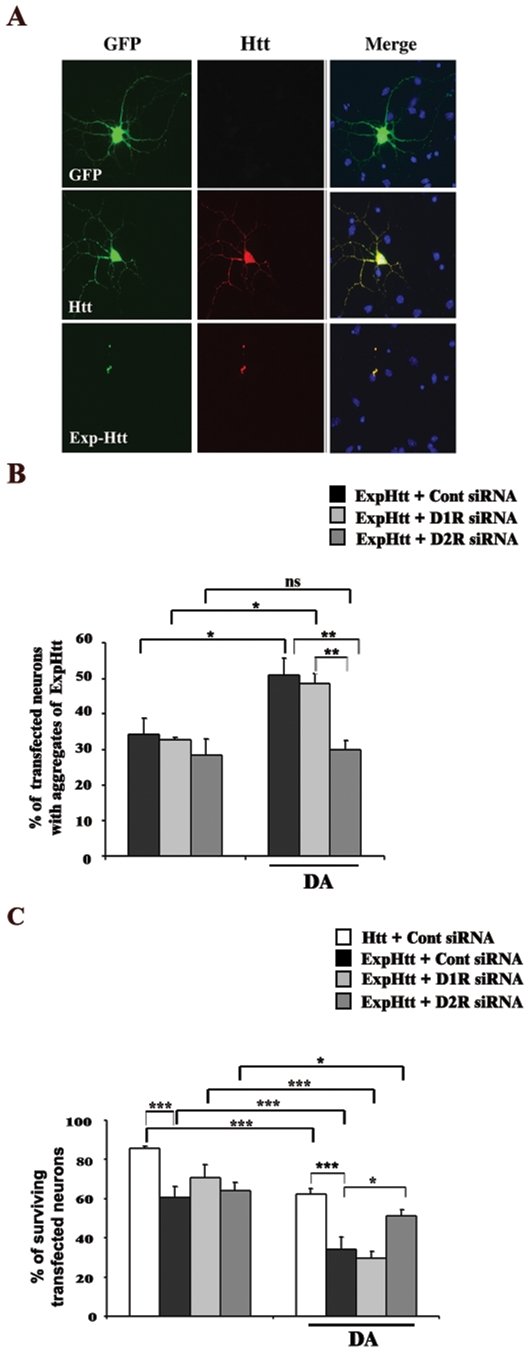
DA-mediated potentiation of aggregates formation and neuronal death induced by ExpHtt in striatal neurons involves D2R but not D1R stimulation. A: Immunocytochemical detection of Htt was performed on striatal neurons transfected with either GFP alone, GFP-tagged Htt or GFP-tagged ExpHtt. Nuclei were labelled using Hoechst (blue). Note that ExpHtt and GFP perfectly colocalize in aggregates. B and C: Seventy two hours after RNA interference procedure, primary neurons were transfected with Htt or ExpHtt and treated with DA (100 µM) for 10 (B) or 24 (C) hours. B: quantification of transfected neurons with aggregates of ExpHtt. C: quantification of surviving transfected neurons based on Hoechst labeling. Data were analyzed from at least 3 independent experiments (100 transfected neurons per condition and per experiment) and expressed as mean±SEM. (***P<0.001, **P<0.005, *P<0.05, ns: non significant).

Using primary cultures of striatal neurons expressing exon 1 of Huntingtin with a polyglutamine stretch (103Q: ExpHtt) fused to EGFP, we recently showed that DA, via D2 receptor stimulation, potentiates aggregate formation and neurodegenerescence induced by ExpHtt. These results were based on pharmacological studies made on primary striatal neurons from WT or knock-out mice for D2 receptors (D2R) [10]. Other *in vitro* studies documented that D1 receptors (D1R) play a role in mutant huntingtin-mediated aggregates formation and apoptosis [23], [24], [25]. In order to clarify the specific contribution of D1R and D2R on DA-mediated potentiation of aggregates formation and neuronal death in our model system, we used molecular tools to abrogate D1R and D2R expression. We first showed expression of both D1R and D2R in primary cultures from E14 striatal neurons after 7 days in vitro (DIV 7) (Fig. 1A–D). Of interest, both the full-length and spliced version of D2 receptors (D2L and D2S) were present in these striatal neurons (Fig. 1B). Specific invalidation of D1R and D2R mRNA and protein expression was achieved following siRNA transfection in striatal neurons (Fig. 1). Then, using this siRNA molecular approach, we focused on the role of both D1and D2 receptors in DA-mediated potentiation of aggregates formation and neuronal death induced by ExpHtt. We took advantages of GFP-tagged cDNAsto visualize expression and localization of Htt and ExpHtt in transfected neurons (Fig. 2A). As a consequence, aggregates of ExpHtt was visualized owing to GFP labelling. Knockdown of D2R (both D2L and D2S) but not D1R, using specific siRNAs resulted in a total inhibition of DA-induced aggregates formation (Fig. 2B), as previously demonstrated from D2R-knock out mice [10]. As expected, D2R siRNA only partially inhibited DA-mediated neuronal death in ExpHtt expressing neurons (Fig. 2C). Furthermore, specific knockdown of D1R did not affect neuronal death in ExpHtt expressing neurons (Fig. 2C). Thus, having shown the specific involvement of D2R but not D1R stimulation in aggregate formation and neuronal death, we used for the following experiments a specific D2R agonist, quinpirole (Quin), to study D2R signalling pathway involved in the potentiation of ExpHtt-induced cellular dysfunctions.

### D2 Receptor Stimulation Potentiates Neuritic Retraction and Growth Cone Collapse Induced by ExpHtt in Striatal Neurons

**Figure 3:**
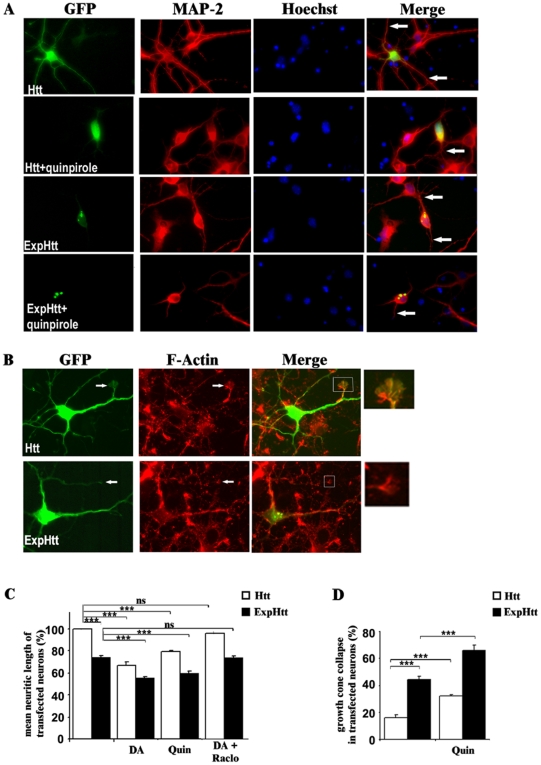
D2 receptor stimulation potentiates neuritic retraction and growth cone collapse induced by ExpHtt in striatal neurons. A) Immunohistochemical detection of MAP2 protein (red) was performed on striatal neurons transfected with either Htt or ExpHtt (GFP) and treated or not for 14 hours with quinpirole (10 µM). Nuclei were labelled using Hoechst (blue). The white arrow in the merge panel points to a neurite in transfected neurons. B) Phalloidin labelling of F-actin was assayed to visualize growth cone on Htt and ExpHtt-expressing striatal neurons. White arrows and insert in the merge panel depict a growth cone in a transfected neuron. C) Quantification of mean neuritic length in transfected neurons was determined from MAP2 staining using ImageJ software in Htt (white bars) and ExpHtt (black bars)-expressing neurons, treated or not with dopamine (DA, 100 µM), quinpirole (Quin, 10 µM) or DA plus raclopride (1 µM) for 14 hours. D) Quantification of growth cone collapse was determined on phalloidin staining in Htt (white bars) and ExpHtt (black bars)- transfected neurons treated or not with quinpirole (10 µM) for 14 hours. C and D) Data were analyzed from at least 3 independent experiments (100 transfected neurons per condition and per experiment) and expressed as mean±SEM.(***P<0.001, ns: non significant).

Once aggregates of ExpHtt are formed, they destabilize microtubules and disrupt the cellular architecture [15]. We first investigated whether ExpHtt had any effect on the dynamic organization of the cytoskeleton and neuritic outgrowth in our model system. Using MAP2 (Microtubule-Associated-protein) immunostaining we analyzed the mean neuritic length of transfected neurons (Fig. 3A, C). Statistical analyses revealed a significant decrease of neuritic length (−25%) in ExpHtt expressing neurons when compared to Htt-transfected cells. We then analyzed a possible potentiation of DA and/or D2-Receptor stimulation on ExpHtt-mediated neuritic retraction. All experiments were performed in the presence of anti-oxidants, in order to avoid indirect effect of DA auto-oxidation. DA and quinpirole treatments potentiated significantly the reduction of neuritic length induced by ExpHtt alone (Fig. 3A, C). Importantly, DA and quinpirole treatments also induced a significant decrease of neuritic length in non-transfected striatal neurons (−30%; data not shown) as well as in Htt-expressing neurons (Fig. 3A, C). In both Htt- and ExpHtt-expressing neurons, DA effects on neuritic retraction were reversed by the selective D2 receptor antagonist, raclopride (Fig. 3C). This indicates that the major effects exerted by DA on neuritic retraction are mediated by D2 receptor stimulation.

Neuritic retraction is accompanied by a disorganization of actin filaments that can be visualized by rhodamine-phalloidin labelling. The filaments of actin are concentrated in filopodes and lamellipodes of growth cones (Fig. 3B). Actin filaments are mainly observed at the tips of the neurites and present very well structured and large growth cones, in most neurons expressing Htt. About 20% of collapse was found in Htt-expressing neurons (Fig. 3B, D). A significant increase of growth cone collapse was observed in ExpHtt-expressing neurons (42% of collapse) (Fig. 3B, D). This collapse was even higher upon quinpirole treatment, in both Htt and ExpHtt transfected neurons (30% and 65%, respectively, Fig. 3D), indicating that D2 receptor stimulation promotes a disorganization of actin filaments.

### D2-R Mediated Neuritic Retraction and Growth Cone Collapse Is Associated with ROCK-II Activation in Striatal Neurons

**Figure 4:**
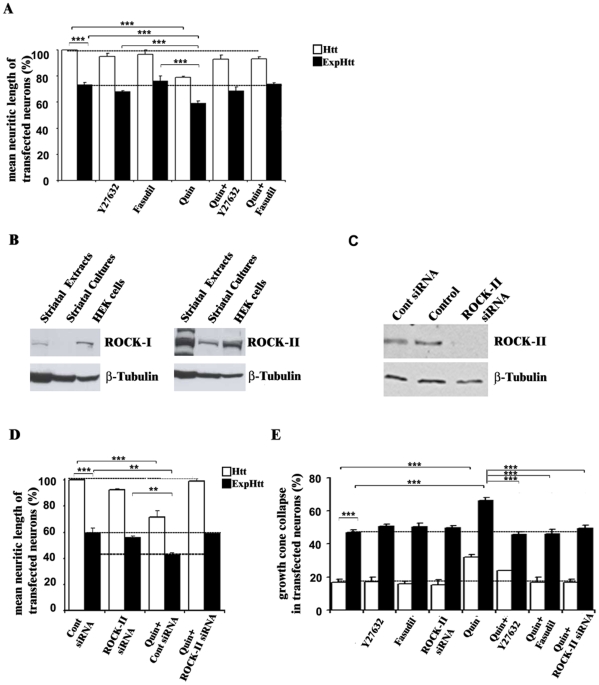
D2-R mediated neuritic retraction and growth cone collapse is associated with ROCK-II activation in striatal neurons. **A**) Quantification of mean neuritic length in neurons transfected with Htt (white bars) or ExpHtt (black bars) was determined from MAP-2 staining. Transfected neurons were pre-treated with ROCK pharmacological inhibitors Y27632 (10 µM) or Hydroxyfasudil (10 µM) 30 minutes prior to and during quinpirole treatment (10 µM) (14 hours). Data are expressed as mean±SEM from 3 independent experiments (100 transfected neurons per condition and per experiment) (***P<0.001). B) Striatal extracts from adult mouse (striatal extracts), striatal neurons in culture (striatal cultures) and HEK293 cells extracts (HEK cells) were processed for Western-blot with specific antibodies against ROCK-I (left) and ROCK-II (right). Similar amounts of proteins were loaded in each case. β-tubulin was used for control loading. Note the high expression level of ROCK-II in both striatal extracts and culture when compared to ROCK-I expression. C) Control siRNA (50 nM) or siRNA targeting ROCK-II (50 nM) were transfected in primary striatal neurons for 72 hours. Extracts from siRNA-transfected or non transfected (control) neurons were processed for Western-blot with specific antibodies against ROCK-II and β-tubulin. Note the knock-down of ROCK-II expression. **D**) Quantification of mean neuritic length in neurons transfected with Htt (white bars) or ExpHtt (black bars) after quinpirole treatment (10 µM) alone or in the presence of control siRNA (50 nM) or ROCK-II siRNA (50 nM). Data are expressed as mean±SEM from 3 independent experiments (100 transfected neurons per condition and per experiment). (***P<0.005, **P<0.05). E) Quantification of growth cone collapse in neurons transfected with Htt (white bars) or ExpHtt (black bars) after quinpirole treatment (10 µM) for 14 hours alone or in the presence of ROCK inhibitors (10 µM) or ROCK-II siRNA (50 nM). Data are expressed as mean±SEM from 3 independent experiments (100 transfected neurons per condition and per experiment). In these experiments, neurons transfected with control siRNA showed no significant difference with non transfected neurons (control) alone (***P<0.001).

Various signalling pathways participate in the dynamic reorganization of the cytoskeleton and neuritic elongation, among which the Rho/ROCK signalling pathway is thought to play a key role [17], [18], [19]. We thus investigated a possible involvement of this pathway in neuritic retraction induced by ExpHtt in the presence or not of the D2 agonist treatment. The ROCK pharmacological inhibitors Y27632 and Hydroxyfasudil were used as a first approach. These inhibitors, applied 30 minutes prior to, and during quinpirole treatment, reversed D2-mediated effects on neuritic retraction in Htt- and ExpHtt- expressing neurons (Fig. 4A). By contrast, they failed to affect neuritic retraction induced by ExpHtt alone (Fig. 4A). We then wished to confirm these observations using siRNAs to abolish ROCK protein expression. Two different genes encode ROCK, giving rise to ROCK-I and ROCK-II. Both proteins are implicated in the formation of stress fibers and suppression of neurite outgrowth and are ubiquitiously expressed, with an enrichment of ROCK-II in the central nervous system [17]. We checked ROCK-I and ROCK-II expression in striata from adult mice (Fig. 4B: striatal extracts), in striatal neurons in culture (Fig. 4B striatal culture), and in HEK cells extracts as a positive control by western blot. Both proteins were found at 160 KDa, their expected molecular weight, in HEK cells (Fig. 4B). Very low levels of ROCK-I were found when compared to ROCK-II in striatal cells both *in vivo* and *in vitro* (Fig. 4B).

Further studies were thus focused on ROCK-II knock-down. Having established the efficiency of siRNAs on ROCK-II expression by western-blot (Fig. 4C), we transfected them (DIV 4) along with cDNAs encoding Htt or ExpHtt (DIV7). The ROCK-II knock-down effects were analyzed on neuritic retraction 14 hours later, in the presence of quinpirole (Fig. 4D). ROCK-II knock-down totally reversed potentiation by quinpirole of ExpHtt-induced neuritic retraction. Similar results were found using specific inhibitors of ROCK, Y27632 and Hydroxyfasudil. By contrast, neither molecular nor pharmacological inhibition of Rho/ROCK signalling reversed neuritic retraction induced by ExpHtt alone. A total reversal of D2-mediating effects was also found on growth cone collapse, without modification of ExpHtt-induced effects (Fig. 4E).

**Figure 5:**
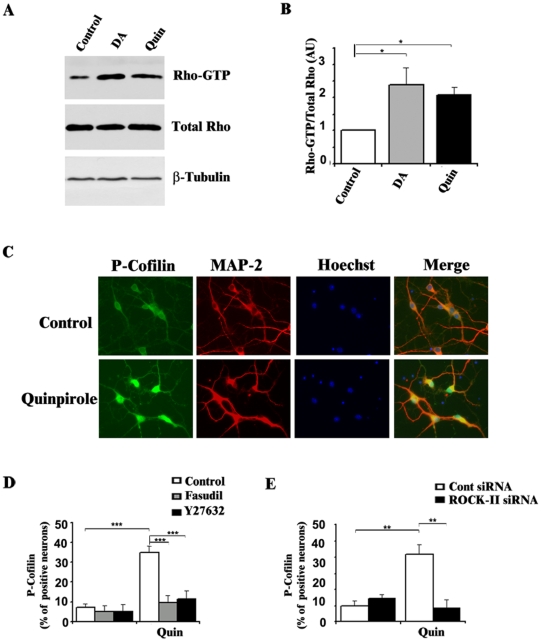
DA and quinpirole activate Rho/ROCK pathway on striatal neurons. A) Western-blot detection of Rho-GTP (top) along with Total Rho (middle) and β-tubulin (bottom) after 15 minutes of treatment with DA (100 µM) and quinpirole (10 µM) in non-transfected striatal neurons. B) Quantification of Rho-GTP activity from 3 independent experiments. Total Rho was expressed as Arbitrary Unit (AU). Note the significant increase of Rho-GTP/Total Rho after 15 minutes of DA (Grey bar) and quinpirole (Black bars) treatment (*P<0.05). C) Immunocytochemical detection of P-Cofilin (green) and MAP2 (red) proteins 20 minutes after quinpirole treatment (10 µM, lower panel) and in Control conditions (upper panel). Nuclei were labelled using Hoechst (blue). D and E) Quantification of P-Cofilin positive neurons (n = 3 independent experiments, 100 neurons per experiment) using Metamorph software after quinpirole treatment alone or in the presence (D) of pharmacological inhibitors of ROCK, Hydroxyfasudil (10 µM) and Y-27632 (10 µM) or (E) after transfection of siRNA ROCK-II. B, D and E, Data are expressed as mean±SEM (***P<0.001, **P<0.005, *P<0.05).

Altogether our data provide a strong, albeit indirect, evidence of a coupling between D2 receptors and Rho/ROCK activation. The DA/D2 receptor subtype has been classically coupled to inhibition of adenylate cyclase [26], but recent evidence in the literature also supported a coupling between D2 receptors and Rho-GTP binding protein [27]. We thus wished to provide a direct evidence of Rho/ROCK activation by D2 receptor stimulation instriatal neurons. Rho activity was measured using the ratio of Rho-GTP levels by comparison to total Rho proteins (Fig. 5A,B). Both DA and quinpirole treatments significantly increased Rho activity after 15 min of treatment. Once activated by Rho, ROCK proteins can phosphorylate several substrates, including LIM kinase which phosphorylates Cofilin at Ser3 [28]. Cofilin critically controls actin filament dynamics and reorganization. Using immunocytochemical approaches, we investigated phosphorylation of cofilin after quinpirole treatments on striatal neurons and found an hyperphosphorylation after 20 minutes of treatment in about 35% of striatal neurons (Fig. 5C–E). This hyperphosphorylation was reversed by ROCK inhibitors (Fig. 5D) and ROCK-II siRNA treatments (Fig. 5E). Altogether these data provide the first evidence that D2 receptor stimulation induces phosphorylation of cofilin via the Rho/ROCK signalling pathway in striatal neurons.

### D2-R Stimulation Potentiates ExpHtt-Induced Aggregate Formation via ROCK-II Activation

**Figure 6:**
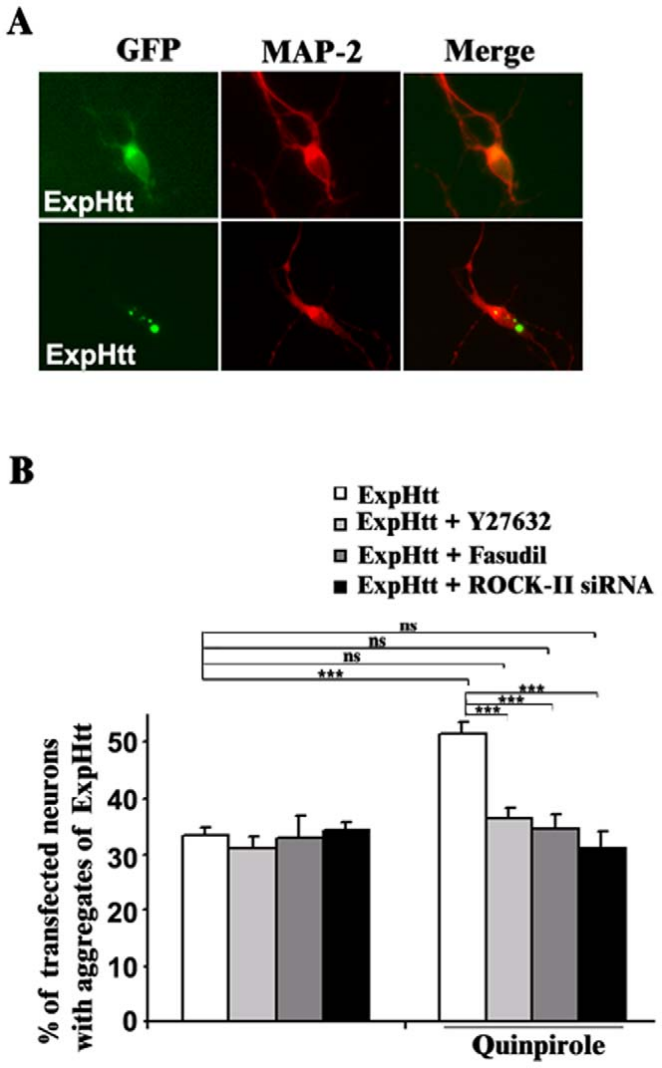
D2-R increases ExpHtt aggregates formation via ROCK-II activation in striatal neurons. A) ExpHtt aggregates detection based on GFP fluorescence in primary striatal neurons transfected with ExpHtt (tagged with GFP) for 10 hours and MAP2 immunostaining (red). Note, at this time point, that ExpHtt present either a diffuse expression pattern (top panel) as well as aggregates formation (bottom panel). B) Quantification of ExpHtt-transfected neurons with aggregates after quinpirole treatment (10 µM) for 10 hours alone or in the presence of pharmacological inhibition of ROCK (Y27632, 10 µM and Hydroxyfasudil, 10 µM) or ROCK-II siRNA (50 nM). Data are expressed as mean±SEM from 3 independent experiments (100 transfected neurons per condition and per experiment) (***P<0.001, ns: non significant).

The pattern of ExpHtt expression into striatal neurons can be observed owing to the GFP tag at the C-terminal region of exon 1 ExpHtt. Thus, ten hours after transfection, ExpHtt shows a diffuse pattern in 65% of transfected neurons (Fig. 6A, upper panel), while aggregates of ExpHtt are observed in the remaining transfected neurons (35%) (Fig. 6A, lower panel). According to our previous observations [10], D2 agonist (quinpirole) treatment significantly increased the number of transfected neurons containing aggregates of ExpHtt (52% versus 35%) (Fig. 6B).

To gain insights into the mechanisms that lead to D2 effects on aggregate formation, we investigated the role of ROCK inhibitors and ROCK siRNAs. D2-mediated aggregate formation was totally reversed by ROCK inhibitors, without any significant effects on aggregates formed by ExpHtt alone. These data were confirmed using ROCK-II siRNAs (Fig. 6B). Thus, our findings indicate that ROCK-II signalling is implicated, downstream D2 receptors, in the potentiation of ExpHtt aggregate formation.

### D2-R Stimulation Potentiates ExpHtt-Induced Neuronal Death via ROCK-II Activation

**Figure 7:**
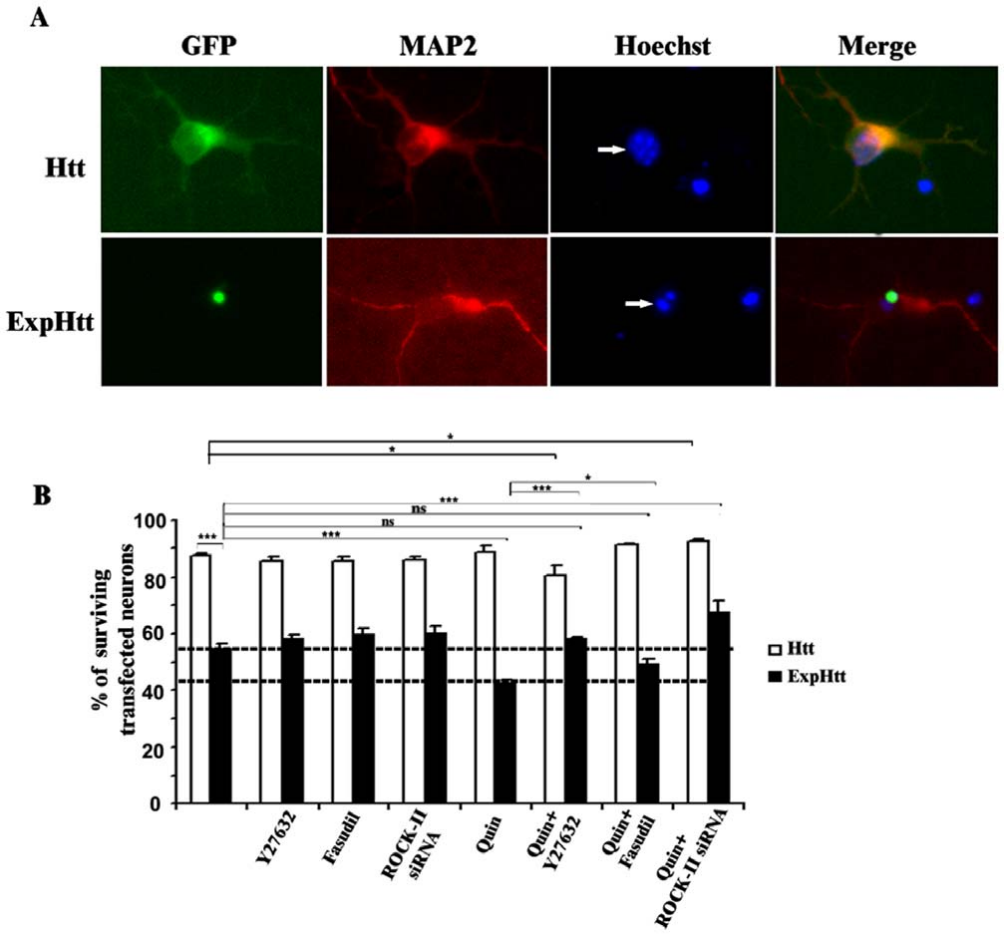
D2-R potentiates striatal neuronal death induced by ExpHtt via ROCK-II activation. A) Immunohistochemical detection of MAP2 protein (red) and nuclei labelling using Hoechst (blue) were performed on striatal neurons transfected for 24 hours with either Htt (top panel) or ExpHtt (bottom panel). The white arrow in the Hoechst panel points to a healthy nucleus (top panel) a fragmented nucleus (bottom planel) in an Htt- and ExpHtt-transfected neuron, respectively. B) Quantification of surviving transfected neurons based on Hoechst labelling. Neurons transfected with Htt (white bars) or ExpHtt (black bars) were treated with quinpirole (10 µM) for 24 hours alone or in the presence of pharmacological inhibition of ROCK (Y27632, 10 µM and Hydroxyfasudil, 10 µM) or ROCK-II siRNA (50 nM). Data are expressed as mean±SEM from 3 independent experiments (100 transfected neurons per condition and per experiment)(***P<0.001, *P<0.05, ns: non significant).

We previously showed [10] that D2 agonist treatment potentiated striatal death induced by ExpHtt. Cell survival was analyzed 24 hours after transfection by appreciation of nucleus morphology (DNA fragmentation) by Hoechst staining (Fig. 7A). Quinpirole significantly potentiated ExpHtt-induced neuronal death without affecting cell survival in Htt-expressing neurons. The ROCK inhibitors (Y-27632 or Hydroxyfasudil) and ROCK-II siRNA totally rescued toxicity induced by the D2 agonist in ExpHtt-expressing striatal neurons with no effects against toxicity induced by ExpHtt itself (Fig. 7B). Altogether, these data indicate that the Rho/ROCK-II signalling pathway is a major pathway downstream D2 receptor for mediating toxicity in ExpHtt-expressing neurons.

## Discussion

In the present work, we wished to analyze how DA *via* DA receptors stimulation might account for aggregate formation and potentiation of neuronal death in ExpHtt expressing striatal neurons. We first confirm our previous results [10], [11] showing that DA potentiation of ExpHtt toxicity is mediated via D2R but not D1R stimulation, in our model system, i.e. in striatal neurons expressing a truncated version of ExpHtt. We show the first evidence for a coupling of D2 receptors to the Rho/ROCK-II signalling pathway. In cultured striatal neurons, inhibition of this pathway reversed DA/D2 receptor-mediated neuritic retraction, growth cone collapse, aggregate formation and neuronal death induced by ExpHtt in the presence of D2 agonist. By contrast with findings in other model systems of HD (Drosophila, and HEK cells), we failed to demonstrate a clear involvement of ROCK in ExpHtt induced cellular phenotypes in striatal neurons.

Neurodegeneration in HD occurs most prominently in the basal ganglia and neocortex. Striatal neurons show a range of vulnerabilities in Huntington's disease. Medium Spiny Neurons (MSNs) are the first affected striatal cells while interneurons are spared, even at a late stage of the disease. Among the MSNs, the GABAergic/enkephalinergic, which preferentially express D2 receptors and project to the external segment of globus pallidus (Gpe) tend to degenenerate before the substance P/dynorphinergic striato-nigral neurons expressing D1 receptors [29], [30], [31]. In HD patients, primates and mouse models of HD, aggregates appear first in the neuropil [9], [14], [16], [32], [33], [34], where they are thought to be toxic by poisoning the axonal transport [11], [16], [35]. The reduction of these aggregates leads to an improvement of the phenotype in HD mice [36]. Interestingly, the Substancia Nigra (SN) and the GPe, which receive afferents from the D2R-expressing striatal neurons, are the first brain areas where neuropil aggregates appear, in mice and human [16], [34], [37], [38]. Hyperdopaminergic HD mice display an accelerated clinical and neuropathological phenotype with early formation of neuropil aggregates that predominate in the GPe [9], which express D2R but not D1R. In primary culture of striatal neurons expressing ExpHtt, D2 receptor stimulation increases the formation of neuropil aggregates, while striatal neurons from D2R knock-out mice fail to show any vulnerability to DA for aggregate formation (11). By contrast, D1R stimulation did not influence the total number of mutant Htt aggregates in SK-N-MC expressing ExpHtt (N-term-103Q) [24]. These observations were strengthened in the present work, where D2R- but not D1R- siRNAs prevented DA-mediated effects on aggregate formation. Thus, altogether, these findings suggest that D2 receptor stimulation plays a key role in GABAergic/MSN vulnerability to ExpHtt, via potentiation of neuropil aggregates and axonal poisoning. Other deleterious effects of D2 receptor stimulation on ExpHtt induced neuronal dysfunctions were recently unraveled (41). In an elegant study, the authors showed that D2 receptor stimulation increased ExpHtt-induced deficiency of mitochondrial complex II protein activity, which plays a key role in oxidative energy metabolism. In cellular models expressing the full length mutant Htt, immortalized mutant STHdh^Q111^, striatal neuronal progenitor cell lines or striatal neurons from YAC128 mice, D1R stimulation but not D2R is involved in DA-mediated cell death and potentiation of glutamate toxicity [23], [25]. In these studies, D1R-mediated effects were critically linked to increased calcium influx. Thus, depending on the expression of the full length or the cleaved fragment of mutant Htt, either D1R or D2R seem to be differentially involved in DA-mediated toxicity on striatal neurons.

In our *in vitro* model system, D2 receptor stimulation increased neuritic retraction and growth cone collapse in mutant- but also normal Htt-expressing neurons. This observation corroborates *in vivo* data showing that D2 receptors stimulation results in pruning of the terminal arbor of substantia nigra pars compacta (SNpc) DA neurons [39], [40]. Furthermore, the genetic invalidation of D2 receptor in mice resulted in a significant increase in terminal tree size of these neurons, as well as the density of varicosities in the caudate-putamen (42,43). We can thus hypothesise that the disorganization of the neuritic arbors described in HD might be, at least in part, due to local D2 receptor stimulation. D2 receptors are classically described as negative regulators of the cAMP/PKA pathway, via coupling to Gαi/o [41]. They can also couple to phospholipase (PLCb) via Gq, mobilize intracellular calcium stores [42] and activate the MAPK/ERK signalling cascade [42], [43]. Dopaminergic stimulation of D2 receptors can also activate or inhibit the Akt phosphorylation level (Thr308), depending on the time of stimulation, short or long, respectively [43], [44]. In the present study, we present the first evidence for a coupling between D2 receptors and the Rho/ROCK signalling pathway. Such coupling was suggested previously, by a study showing that D2-mediated activation of Phospholipase D (PLD) was abolished by preincubation of the cells with C3 exoenzyme, a specific inhibitor of the Rho proteins [27]. Other G protein-coupled receptors have been described for their ability to activate Rho proteins. The classical activator of this pathway is the lysophosphatidic acid (LPA) receptor which acts through G12/G13 to activate RhoA and ROCK and lead to neuritic retraction and growth cone collapse [45], [46], [47] The details of the mechanism by which D2 receptor stimulates Rho proteins remain unclear but could involve the scaffolding proteins β-arrestin that have been associated with the activation of Rho downstream Angiotensin A2 receptors [48] and are known to be critical for D2 receptor internalization [49].

ROCK proteins are principal mediators of RhoA activity in the nervous system where they have been largely reported as regulators of neuritic length and growth cone collapse [50]. Screening for biologically active molecules that inhibit polyglutamine aggregation, lead to the identification of a pharmacological ROCK inhibitor, Y-27632 [21], [22] When Y-27632 was administrated in a *Drosophila* model of HD, it reduced Htt-exon1 (93Q) induced neurodegeneration. In HEK cell line expressing Htt exon1 (72Q), it was necessary to knock-down both ROCK-I and Protein Kinase C-related protein (PRK2) proteins to abolish polyglutamine aggregation [21] In Neuro2a cells expressing Htt-exon1 (150Q), knock-down of ROCK-II and ROCK-I reduced aggregation [51]. In striatal neurons, *in vitro* and *in vivo*, we found a poor expression of ROCK-I, while ROCK-II was strongly expressed. Global inhibitors of ROCK or knock-down of ROCK-II expression, failed to reverse neuronal dysfunctions induced by ExpHtt by itself. However, the pharmacological inhibitor of ROCK, as well as ROCK-II-siRNAs, reversed D2-mediated effects on ExpHtt-induced phenotype. We thus propose that, in striatal neurons, the Rho/ROCK-II signalling pathway is critically involved in D2-mediated vulnerability in HD. Interestingly, ROCK proteins have been proposed as a promising therapeutic target in various neurological conditions [52]. In the CNS, inhibition of ROCK using Y27632 promotes axon growth after spinal cord injury in experimental animal models [53] and reduces the expression of amyloid-βpeptide in a mouse model of Alzheimer's disease [54].

Because long term pharmacological blockade of D2 receptors may be poorly tolerated in patients [55], [56], the inclusion of Rho/ROCK inhibitors could be an interesting therapeutic option to target the vulnerability of D2-R expressing striatal neurons in HD.

## Materials and Methods

### Mouse Husbandry

Mice were housed in a temperature-controlled room maintained on a light/dark cycle. Food and water were available *ad libitum*. Experiments were performed in accordance with standard ethical guidelines (U.S. National Institutes of Health publication n° 85-23, revised 1985, and the European Committee Guidelines on the Care and Use of Laboratory Animals directive 86/609/EEC).

### Primary Striatal Culture and Drug Treatment

Primary striatal neurons were dissected out from 14 days old embryos from pregnant Swiss mice (Janvier, Le Genest Saint Isle, France) as previously described [43]. After dissociation, cell pellets were resuspended in Neurobasal medium supplemented with B27 (Invitrogen), 500 nM L-glutamine, 60 µg/ml Pennicilin-streptomycin and 25 µM β-mercapto-ethanol and then plated on poly-L-lysine-coated 24-well (1,33×10^5^ cells by well) or 6-well plates (6,321×10^5^ cells by well) at 37°C in a humidified 5% CO_2_ incubator. Using this preparation, the cell culture contains in majority cells from neuronal and not glial origin.

After 7 days in culture, neurons grown in 24 well plates, were transiently transfected with GFP-tagged Htt constructs (provided by HDF Resource Bank, UCLA) encoding the first exon of human Htt containing either 25 (Htt) or 103 (ExpHtt) continuous CAA or CAG repeats. The sequence encoding an enhanced Green Fluorescence Protein (GFP) was inserted in frame at the C-terminus of each construct. Three hours and half after transfection, the medium was removed and replaced by the complete Neurobasal Medium containing dopamine (DA, 100 µM, Calbiochem), quinpirole (Quin, 10 µM RBI). Cells were then replaced at 37°C for the appropriate time. For the pharmacological treatments, Y-27632, (10 µM; Tocris) Hydroxyfasudil (10 µM; Sigma) or Raclopride (Raclo, 1 µM Sigma) were added 30 min before and during quinpirole treatment.

### RNA Interference

SiRNAs targeted dopamine D1 (D1R), both isoforms of dopamine D2 (D2R) receptors, D2Long and D2Short (D2LR and D2SR, respectively) and ROCK-II were designed to specifically interfere with dopamine D1R, D2R and ROCK-II expression. The target sequences were as follows: D1R, 5′-CCUGGAAGAUGCCGAGGAU-3′; D2R, 5′-CCAGAGAGGACCCGGUAUA-3′ and ROCK-II, 5′-CAAUGAAGCUUCUUAGUAA-3′ (Eurogentec). Neurons were transfected at day *in vitro* 4 (DIV) with 50 nM of siRNA against D1R, D2R and ROCK-II or siRNA control (Cont siRNA #2, Ambion). After 72 hours of silencing, primary neurons were used for transfections experiments and drug treatment.

### RNA Isolation and RT-PCR

Seventy two hours after transfection with D1R or D2R siRNAs, total mRNA was isolated using RNeasy mini kit (Qiagen) following the manufacturer's instructions. Reverse transcription was performed using SuperScript III (Invitrogen) followed by cDNA amplification with specific primers (Eurogentec S.A.). The nucleotide sequences of the primers were as follows: D1R, forward 5′-TTCTTCCTGGTATGGCTTGG-3′, reverse 5′-GCTTAGCCCTCACGTTCTTG-3′; D2LR, forward 5′-AACTGTACCCACCCTGAGGA-3′, reverse 5′-GTTGCTATGTAGACCGTG -3′ and D2SR, forward 5′-CACCACTCAAGGATGCTGCCCG-3′, reverse 5′-GTTGCTATGTAGACCGTG -3′. Amplification of Arginin Rich Protein (ARP), used as an housekeeping gene, was performed using the following primers forward 5′-GAACGTCGTCTTCGTGTTCA-3′, reverse 5′- AAAACCTGGACGAAGGAGGT -3′.

### Immunocytochemistry and Histological Analysis

Striatal neurons were fixed in 2% paraformaldehyde in PBS for 40 minutes at room temperature and permeabilized with Methanol/Acetone (1∶1 ratio) for 10 min at 4°C. After washing with PBS, cells were preincubated with blocking buffer (10% Normal Goat Serum in PBS) at room temperature for 1 h and then incubated with primary antibody in PBS overnight at 4°C: mouse anti-microtubule-associated protein 2 (1/1000, Sigma), rabbit anti-P-Cofilin (1/500, Cell Signaling,) and mouse anti-Huntingtin (1/1000, Chemicon). Cells were rinsed with PBS and incubated for 2 h at room temperature with the secondary antibody, Cy3-conjugated anti-mouse IgG (1/2000,Jackson Laboratories) or Rabbit-FITC (1/200, Sigma). To analyze the nuclei morphology, cells were stained with Hoechst (1/20000 in PBS) for 5 min at room temperature, followed by three PBS washes. Aggregates of ExpHtt were visualized by GFP staining. The cells were mounted under cover slips using a Vectashield medium (Vector Laboratories) and examined with a Leica DM4000B fluorescence microscope (X40). The percentage of transfected neurons containing aggregates of Exp-Htt were determined by counting neurons containing aggregated GFP versus diffuse GFP. Neurons containing condensed or fragmented nuclei were scored as dying cells. The neuritic length was evaluated by tracing the neurites using the software ImageJ (version 1.34 s). The actin cytoskeleton staining was performed by incubating fixed cells with phalloïdine TRITC (1/500) diluted in PBS-2% BSA for 20 min at room temperature. Each experiment was conducted in triplicate, and at least 100 transfected neurons were randomly analyzed in each experiment.

### Cell Extracts and Western Blot Analysis

Samples were prepared from neurons and HEK293 cells homogenized in a boiling lysis buffer containing 1% SDS and 1 mM sodium orthovanadate and briefly sonicated. Equal amounts of proteins were resolved by SDS-PAGE gels and transfered to PVDF membrane (Amersham). After blocking in 5% nonfat dry milk (for ROCK-I and ROCK-II) or 5% BSA (Bovine Serum Albumin, SIGMA) (for D1R and D2R immunobloting) in TBS for 1 h at room temperature, membranes were incubated overnight at 4°C with primary antibodies, rabbit polyclonal anti-ROCK-I anti-ROCK-II (1/500, Santa Cruz), mouse monoclonal anti-D1R (1/250, Millipore), rabbit polyclonal anti-D2R (1/1000, Millipore) and mouse monoclonal anti-β-tubulin (1/5000,Sigma), and revealed with appropriate anti-rabbit or anti-mouse peroxidase-conjugated secondary antibodies (1/5000, Jackson ImmunoResearch Laboratories) and the enhanced chemiluminescent (ECL) detection system (Pierce).

### Rho Activation Assay

Rho activation was analyzed using Rho assay kit (Upstate Biotechnology). After DA (100 µM) and quinpirole (10 µM) treatments for 15 min, cells were lysed in 50 mM Tris-HCl, pH 7,5, 150 mM NaCl, 2 mM EDTA, 1 mM sodium orthovanadate, 50 mM MgCl_2_, 0,1% SDS, 1% Nonidet P-40, 1% sodium deoxycholate and complete protease inhibitor cocktail (Sigma) for 15 min at 4°C. The Rho active form, Rho-GTP, was specifically immunoprecipitated from these lysates incubating with Rho Assay Reagent (Rhotekin fused to the Rho Binding Domain on agarose beads) for 45 min at 4°C on a rotator. After a short spin, the pellet beads were washed three times with lysis buffer and resuspended in 25 µl of 2X Laemmli buffer. Rho-GTP activated bound to rhotekin-agarose beads was subjected to 15% SDS-PAGE, followed by Western blotting with mouse monoclonal anti-Rho (-A,-B,-C) antibody (1/250, Upstate).

### Statistical Analysis

All data were statistically analyzed using GraphPad Prism (GrahPad Software Inc., San Diego,CA). Two-way ANOVA followed by *post hoc* Bonferroni's test was carried out and for all statistical analyses the difference between comparisons was considered to be significant when P<0.05.

## References

[1] Vonsattel JP, Myers RH, Stevens TJ, Ferrante RJ, Bird ED (1985). Neuropathological classification of Huntington's disease.. J Neuropathol Exp Neurol.

[2] Roze E, Saudou F, Caboche J (2008). Pathophysiology of Huntington's disease: from huntingtin functions to potential treatments.. Curr Opin Neurol.

[3] Cass WA (1997). Decreases in evoked overflow of dopamine in rat striatum after neurotoxic doses of methamphetamine.. J Pharmacol Exp Ther.

[4] Vonsattel JP, DiFiglia M (1998). Huntington disease.. J Neuropathol Exp Neurol.

[5] Bowyer JF, Clausing P, Schmued L, Davies DL, Binienda Z (1996). Parenterally administered 3-nitropropionic acid and amphetamine can combine to produce damage to terminals and cell bodies in the striatum.. Brain Res.

[6] Luo Y, Umegaki H, Wang X, Abe R, Roth GS (1998). Dopamine induces apoptosis through an oxidation-involved SAPK/JNK activation pathway.. J Biol Chem.

[7] Ricaurte GA, Guillery RW, Seiden LS, Schuster CR, Moore RY (1982). Dopamine nerve terminal degeneration produced by high doses of methylamphetamine in the rat brain.. Brain Res.

[8] Cyr M, Beaulieu JM, Laakso A, Sotnikova TD, Yao WD (2003). Sustained elevation of extracellular dopamine causes motor dysfunction and selective degeneration of striatal GABAergic neurons.. Proc Natl Acad Sci U S A.

[9] Cyr M, Sotnikova TD, Gainetdinov RR, Caron MG (2006). Dopamine enhances motor and neuropathological consequences of polyglutamine expanded huntingtin.. Faseb J.

[10] Charvin D, Vanhoutte P, Pages C, Borrelli E, Caboche J (2005). Unraveling a role for dopamine in Huntington's disease: the dual role of reactive oxygen species and D2 receptor stimulation.. Proc Natl Acad Sci U S A.

[11] Charvin D, Roze E, Perrin V, Deyts C, Betuing S (2007). Haloperidol protects striatal neurons from dysfunction induced by mutated huntingtin in vivo.. Neurobiol Dis.

[12] Graveland GA, Williams RS, DiFiglia M (1985). Evidence for degenerative and regenerative changes in neostriatal spiny neurons in Huntington's disease.. Science.

[13] Ferrante RJ, Kowall NW, Richardson EP (1991). Proliferative and degenerative changes in striatal spiny neurons in Huntington's disease: a combined study using the section-Golgi method and calbindin D28k immunocytochemistry.. J Neurosci.

[14] DiFiglia M, Sapp E, Chase KO, Davies SW, Bates GP (1997). Aggregation of huntingtin in neuronal intranuclear inclusions and dystrophic neurites in brain.. Science.

[15] Trushina E, Heldebrant MP, Perez-Terzic CM, Bortolon R, Kovtun IV (2003). Microtubule destabilization and nuclear entry are sequential steps leading to toxicity in Huntington's disease.. Proc Natl Acad Sci U S A.

[16] Li H, Li SH, Yu ZX, Shelbourne P, Li XJ (2001). Huntingtin aggregate-associated axonal degeneration is an early pathological event in Huntington's disease mice.. J Neurosci.

[17] Riento K, Ridley AJ (2003). Rocks: multifunctional kinases in cell behaviour.. Nat Rev Mol Cell Biol.

[18] Nakayama AY, Luo L (2000). Intracellular signaling pathways that regulate dendritic spine morphogenesis.. Hippocampus.

[19] Amano M, Ito M, Kimura K, Fukata Y, Chihara K (1996). Phosphorylation and activation of myosin by Rho-associated kinase (Rho-kinase).. J Biol Chem.

[20] Shao J, Welch WJ, Diprospero NA, Diamond MI (2008). Phosphorylation of Profilin by ROCK1 Regulates Polyglutamine Aggregation.. Mol Cell Biol.

[21] Shao J, Welch WJ, Diamond MI (2008). ROCK and PRK-2 mediate the inhibitory effect of Y-27632 on polyglutamine aggregation.. FEBS Lett.

[22] Pollitt SK, Pallos J, Shao J, Desai UA, Ma AA (2003). A rapid cellular FRET assay of polyglutamine aggregation identifies a novel inhibitor.. Neuron.

[23] Paoletti P, Vila I, Rife M, Lizcano JM, Alberch J (2008). Dopaminergic and glutamatergic signaling crosstalk in Huntington's disease neurodegeneration: the role of p25/cyclin-dependent kinase 5.. J Neurosci.

[24] Robinson P, Lebel M, Cyr M (2008). Dopamine D1 receptor-mediated aggregation of N-terminal fragments of mutant huntingtin and cell death in a neuroblastoma cell line.. Neuroscience.

[25] Tang TS, Chen X, Liu J, Bezprozvanny I (2007). Dopaminergic signaling and striatal neurodegeneration in Huntington's disease.. J Neurosci.

[26] Stoof JC, Kebabian JW (1981). Opposing roles for D-1 and D-2 dopamine receptors in efflux of cyclic AMP from rat neostriatum.. Nature.

[27] Senogles SE (2000). The D2s dopamine receptor stimulates phospholipase D activity: a novel signaling pathway for dopamine.. Mol Pharmacol.

[28] Heredia L, Helguera P, de Olmos S, Kedikian G, Sola Vigo F (2006). Phosphorylation of actin-depolymerizing factor/cofilin by LIM-kinase mediates amyloid beta-induced degeneration: a potential mechanism of neuronal dystrophy in Alzheimer's disease.. J Neurosci.

[29] Reiner A, Albin RL, Anderson KD, D'Amato CJ, Penney JB (1988). Differential loss of striatal projection neurons in Huntington disease.. Proc Natl Acad Sci U S A.

[30] Yohrling GJt, Jiang GC, DeJohn MM, Miller DW, Young AB (2003). Analysis of cellular, transgenic and human models of Huntington's disease reveals tyrosine hydroxylase alterations and substantia nigra neuropathology.. Brain Res Mol Brain Res.

[31] Sapp E, Ge P, Aizawa H, Bird E, Penney J (1995). Evidence for a preferential loss of enkephalin immunoreactivity in the external globus pallidus in low grade Huntington's disease using high resolution image analysis.. Neuroscience.

[32] Gutekunst CA, Li SH, Yi H, Mulroy JS, Kuemmerle S (1999). Nuclear and neuropil aggregates in Huntington's disease: relationship to neuropathology.. J Neurosci.

[33] Yang SH, Cheng PH, Banta H, Piotrowska-Nitsche K, Yang JJ (2008). Towards a transgenic model of Huntington's disease in a non-human primate.. Nature.

[34] Tallaksen-Greene SJ, Crouse AB, Hunter JM, Detloff PJ, Albin RL (2005). Neuronal intranuclear inclusions and neuropil aggregates in HdhCAG(150) knockin mice.. Neuroscience.

[35] Lee WC, Yoshihara M, Littleton JT (2004). Cytoplasmic aggregates trap polyglutamine-containing proteins and block axonal transport in a Drosophila model of Huntington's disease.. Proc Natl Acad Sci U S A.

[36] Wang CE, Zhou H, McGuire JR, Cerullo V, Lee B (2008). Suppression of neuropil aggregates and neurological symptoms by an intracellular antibody implicates the cytoplasmic toxicity of mutant huntingtin.. J Cell Biol.

[37] Albin RL, Reiner A, Anderson KD, Dure LSt, Handelin B (1992). Preferential loss of striato-external pallidal projection neurons in presymptomatic Huntington's disease.. Ann Neurol.

[38] Albin RL, Young AB, Penney JB, Handelin B, Balfour R (1990). Abnormalities of striatal projection neurons and N-methyl-D-aspartate receptors in presymptomatic Huntington's disease.. N Engl J Med.

[39] Parish CL, Finkelstein DI, Drago J, Borrelli E, Horne MK (2001). The role of dopamine receptors in regulating the size of axonal arbors.. J Neurosci.

[40] Parish CL, Stanic D, Drago J, Borrelli E, Finkelstein DI (2002). Effects of long-term treatment with dopamine receptor agonists and antagonists on terminal arbor size.. Eur J Neurosci.

[41] Missale C, Nash SR, Robinson SW, Jaber M, Caron MG (1998). Dopamine receptors: from structure to function.. Physiol Rev.

[42] Yan Z, Feng J, Fienberg AA, Greengard P (1999). D(2) dopamine receptors induce mitogen-activated protein kinase and cAMP response element-binding protein phosphorylation in neurons.. Proc Natl Acad Sci U S A.

[43] Brami-Cherrier K, Valjent E, Garcia M, Pages C, Hipskind RA (2002). Dopamine induces a PI3-kinase-independent activation of Akt in striatal neurons: a new route to cAMP response element-binding protein phosphorylation.. J Neurosci.

[44] Beaulieu JM, Sotnikova TD, Marion S, Lefkowitz RJ, Gainetdinov RR (2005). An Akt/beta-arrestin 2/PP2A signaling complex mediates dopaminergic neurotransmission and behavior.. Cell.

[45] Jalink K, Eichholtz T, Postma FR, van Corven EJ, Moolenaar WH (1993). Lysophosphatidic acid induces neuronal shape changes via a novel, receptor-mediated signaling pathway: similarity to thrombin action.. Cell Growth Differ.

[46] Jalink K, van Corven EJ, Hengeveld T, Morii N, Narumiya S (1994). Inhibition of lysophosphatidate- and thrombin-induced neurite retraction and neuronal cell rounding by ADP ribosylation of the small GTP-binding protein Rho.. J Cell Biol.

[47] Kranenburg O, Poland M, van Horck FP, Drechsel D, Hall A (1999). Activation of RhoA by lysophosphatidic acid and Galpha12/13 subunits in neuronal cells: induction of neurite retraction.. Mol Biol Cell.

[48] Barnes WG, Reiter E, Violin JD, Ren XR, Milligan G (2005). beta-Arrestin 1 and Galphaq/11 coordinately activate RhoA and stress fiber formation following receptor stimulation.. J Biol Chem.

[49] Macey TA, Gurevich VV, Neve KA (2004). Preferential Interaction between the dopamine D2 receptor and Arrestin2 in neostriatal neurons.. Mol Pharmacol.

[50] Hirose M, Ishizaki T, Watanabe N, Uehata M, Kranenburg O (1998). Molecular dissection of the Rho-associated protein kinase (p160ROCK)-regulated neurite remodeling in neuroblastoma N1E-115 cells.. J Cell Biol.

[51] Bauer PO, Wong HK, Oyama F, Goswami A, Okuno M (2009). Inhibition of rho-kinases enhances the degradation of mutant huntingtin.. J Biol Chem.

[52] Wettschureck N, Offermanns S (2002). Rho/Rho-kinase mediated signaling in physiology and pathophysiology.. J Mol Med.

[53] Bito H, Furuyashiki T, Ishihara H, Shibasaki Y, Ohashi K (2000). A critical role for a Rho-associated kinase, p160ROCK, in determining axon outgrowth in mammalian CNS neurons.. Neuron.

[54] Zhou Y, Su Y, Li B, Liu F, Ryder JW (2003). Nonsteroidal anti-inflammatory drugs can lower amyloidogenic Abeta42 by inhibiting Rho.. Science.

[55] Adam OR, Jankovic J (2008). Symptomatic treatment of Huntington disease.. Neurotherapeutics.

[56] Correll CU (2007). Acute and long-term adverse effects of antipsychotics.. CNS Spectr.

